# Branch-site occlusion sign predicts the embolic origin of acute ischemic stroke: a meta-analysis

**DOI:** 10.3389/fneur.2023.1139756

**Published:** 2023-06-07

**Authors:** Xinzhao Jiang, Zongjie Shi, Peng Wang, Xu Wang, Fang Liu

**Affiliations:** Center for Rehabilitation Medicine, Department of Neurology, Zhejiang Provincial Peoples Hospital (Affiliated People's Hospital, Hangzhou Medical College), Hangzhou, Zhejiang, China

**Keywords:** branch-site occlusion sign, ILVO-AIS, endovascular therapy, stentriever, neurology

## Abstract

**Objective:**

The study aimed to investigate whether branch-site occlusion (BSO) sign could predict the etiology of acute intracranial large artery occlusion (ILVO) and the stentriever (SR) response.

**Methods:**

We systematically reviewed studies that evaluated the predictive role of BSO for the etiology of ILVO-AIS or EVT outcome between 1 January 2000 and 31 August 2022 from PubMed, Embase, and Web of Science.

**Results:**

The sensitivity and specificity of BSO sign predicting etiology of ILVO-AIS were 0.87 (95% CI 0.81–0.91) and 0.64 (95% CI 0.33–0.87), respectively. The sensitivity and specificity of BSO sign predicting stentriever response were 0.84 (95% CI 0.63–0.94) and 0.61 (95% CI 0.18–0.92), respectively.

**Conclusion:**

The BSO sign could be a valid and precise imaging marker to predict embolism caused ILVO-AIS and recanalization success by SR without rescue therapy.

## 1. Introduction

Acute ischemic stroke (AIS) caused by intracranial large vessel occlusion (ILVO) is a severe and life-threatening disease (1, 2). Endovascular therapy (EVT) is an effective treatment for patients who fail intravenous thrombolysis that may improve prognosis in patients with ILVO-AIS (3, 4). Nevertheless, EVT is not successful in all patients. The possible causes of recanalization failure are different etiologies of ILVO, such as intracranial atherosclerosis (ICAS) and embolism (5). Therefore, it is helpful for identifying the cause of occlusion before performing EVT (3, 5, 6). However, in clinical practice, it is difficult to distinguish between ICAS-AIS and embolism-AIS. Along with the evolution of EVT techniques, intra-procedural angiographic signs have been demonstrated in previous studies and may help to understand the potential etiologies of ILVO-AIS (7). Thus, for accurate and timely selection of EVT strategies, focus should be given to intra-procedural angiographic signs.

Recently, several studies have shown that the branching-site occlusion (BSO) sign in large intracranial arteries can be used as an intra-procedural angiographic sign for predicting embolic occlusion (5, 8, 9). The BSO sign is identified as ILVO with at least one of the following three angiographic signs: (1) anterior communicating artery collateral flow that could not advance to the contralateral internal or middle cerebral artery because the internal cerebral artery bifurcation site was involved (T-occlusion); (2) direct visualization of a Y-shaped or T-shaped filling defect involving a branching site (Y- or T-shaped clot); and (3) another branch could not be seen or was only partially seen when the stentriever was deployed up to one branch across the occlusion site (9). Furthermore, previous studies also showed that the BSO sign can predict the procedural success of the stentriever (SR) without rescue therapy (RT), which has been an effective EVT strategy for ILVO-AIS (9, 10). However, results regarding the predictive value of the BSO sign for the procedural success of the SR without RT varied across studies. Therefore, we performed this meta-analysis to estimate the predictive value of the BSO sign for embolic-related ILVO or AIS (embolic-ILVO/AIS) and the procedural success of the SR without RT in this study.

## 2. Methods

### 2.1. Search strategy

We implemented the meta-analysis according to PRISMA guidelines (11). Eligible studies must have a clear definition of the BSO sign and ILVO-AIS. A BSO sign was identified as the direct visualization of a Y-shaped or T-shaped filling defect involving a branching site (9). ILVO-AIS was defined as a stroke caused by occlusion of the internal carotid artery, or middle cerebral arteries, M1 and M2, verified by vascular exams (12).

We systematically searched PubMed, Embase, and Web of Science for studies that assessed the BSO for predicting the etiology of ILVO-AIS or EVT outcome between 1 January 2000 and 31 August 2022. The search language was limited to English. The search strategy is available in Table 1.

**Table 1:** Detailed search strategy.

**Database**	**Query**	**Results**
PubMed	#1: (((((intracranial atherosclerotic stenosis[Title/Abstract]) OR (intracranial artery stenosis[Title/Abstract])) OR (intracranial Atherosclerosis [Title/Abstract])) OR (intracranial atherosclerotic Disease[Title/Abstract])) OR (ICAS[Title/Abstract]))	121
	#2: (((large vessel occlusion [Title/Abstract]) OR (large artery occlusion [Title/Abstract])) OR (acute stroke [Title/Abstract])) OR (intracranial embolism [Title/Abstract]) OR (acute ischemic stroke [Title/Abstract]))	
	#3: ((thrombectomy [Title/Abstract]) OR (endovascular therapy [Title/Abstract])) OR (endovascular treatment [Title/Abstract])	
	#4:(#1) AND (#2) AND (#3)	
Embase	#1:‘intracranial atherosclerotic stenosis':ab,ti or ‘intracranial artery stenosis':ab,ti or ‘intracranial atherosclerosis':ab,ti or ‘intracranial atherosclerotic disease':ab,ti or ‘ICAS':ab,ti	112
	#2:‘large vessel occlusion':ab,ti or ‘large artery occlusion':ab,ti or ‘intracranial embolism':ab,ti or ‘acute stroke':ab,ti or ‘acute ischemic stroke':ab,ti	
	#3: ‘thrombectomy':ab,ti or 'endovascular therapy':ab,ti or ‘endovascular treatment':ab,ti	
	#4: (#1) AND (#2) AND (#3)	
Web of sciences	#1: (((((((((((TI=(intracranial atherosclerotic stenosis)) OR TI=(intracranial artery stenosis)) OR TI=(intracranial atherosclerosis)) OR TI=(intracranial atherosclerotic disease)) ORTI=(ICAS)) OR TI=(ICAD)) OR AB=(intracranial atherosclerotic stenosis)) OR AB=(intracranial artery stenosis)) ORAB=(intracranial atherosclerosis)) OR AB=(intracranial atherosclerotic disease)) OR AB=(ICAS))	320
	#2: (((((((TI= (large vessel occlusion)) OR TI= (large artery occlusion)) OR TI= (intracranial embolism)) OR TI= (acute stroke)) OR AB= (large vessel occlusion)) OR AB= (large artery occlusion))OR AB= (intracranial embolism)) OR AB= (acute stroke) OR AB= (acute ischemic stroke)	
	#3: (((((TI=(thrombectomy)) OR TI= (endovascular therapy)) ORTI= (endovascular treatment)) OR AB=(thrombectomy)) OR AB= (endovascular therapy)) OR AB= (endovascular treatment)	
	#4:((#1) AND #2) AND #3	

### 2.2. Selection criteria

The inclusion criteria of this study were as follows: (1) studies investigating the association between the BSO sign and outcomes; (2) etiology of LVO-AISs treated with EVT; and (3) studies that provided sufficient information for the construction of the 2 × 2 contingency table (i.e., true and false positives and negatives were provided). Reviews, correspondences, case reports, expert opinions, editorials, and studies with incomplete data were all excluded from the analysis.

### 2.3. Endpoints

(1) *Etiology*: Stroke etiologies were defined as ICAS or embolism according to the criteria used among the included studies (3, 9, 13).
(2) *Therapeutic outcomes:* They are defined as recanalization success (RS) by SR without RT. RS was defined as mTICI 2b-3 (9, 10, 14).

### 2.4. Data extraction and quality assessment

Two investigators independently assessed the included studies and extracted data. Data for the etiology and therapeutic outcomes were extracted to calculate true and false positive and negative rates. We assessed the quality of included studies according to the Quality Assessment of Diagnostic Accuracy Studies-2 (QUADAS-2) by RevMan 5.4 software. The QUADAS-2 consists of four parts, each containing multiple items. The response options for each item were yes, no, and unclear (15).

### 2.5. Statistical analysis

2 × 2 tables were tabulated through true- and false-positives and true- and false-negatives for evaluating the predictive value of the BSO sign for the etiology of ILVO-AIS. We used the numbers to calculate sensitivity and specificity with corresponding 95% confidence intervals (CI). Data were synthesized using a bivariate meta-analysis with a random-effects model developed for synthesis. The meta-analysis was performed by calculating the pooled sensitivity, specificity, positive likelihood ratio, negative likelihood ratio, diagnostic odds ratio, and incidence of RS after SR. To graphically present the results, we plotted the hierarchical summary receiver operating characteristic curves and calculated the area under the curve with its 95% CI by the MIDAS module for STATA (version 15.1) (16). Statistical significance was defined as a *p*-value of ≤ 0.05. We calculated *I*^2^ to assess the degree of heterogeneity across studies. Heterogeneity was assessed by meta-regression. Publication bias was assessed by funnel plot.

## 3. Results

### 3.1. Search results

**Figure 1:**
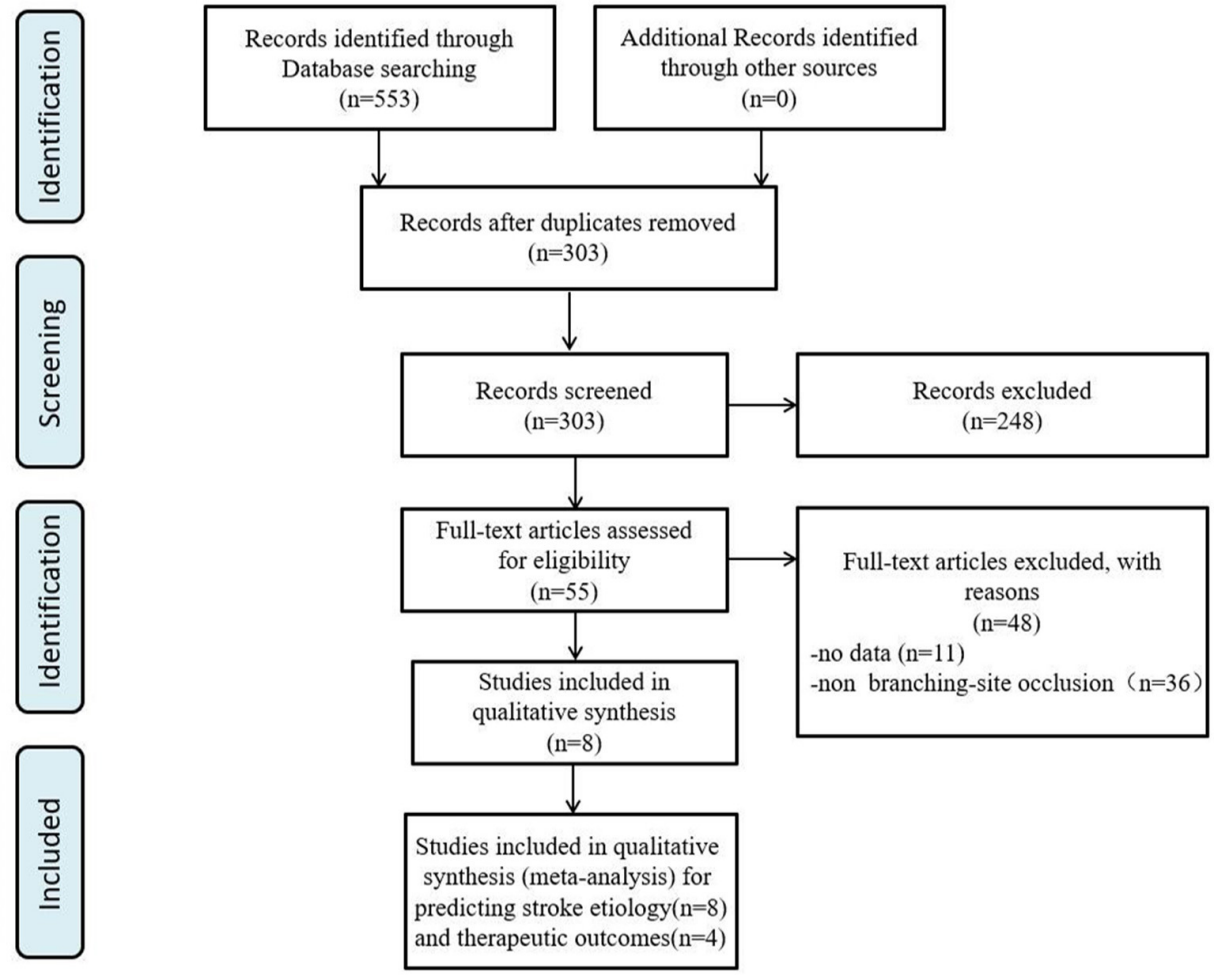
PRISMA flow diagram for study selection.

We identified 553 articles through database searches. After removing duplicates (250) and studies that did not examine the association between imaging signs with ILVO-AIS (*n* = 248), 55 articles remained for further analysis. Of these, 48 articles were excluded: 12 studies provided insufficient raw data, and the other 36 studies assessed imaging signs other than the BSO sign. We finally obtained a total of seven studies for analysis (Figure 1).

### 3.2. Study characteristics

A total of seven studies (1,017 patients) were included. All results, except one, demonstrated the relationship between the BSO sign and the etiology of ILVO-AIS. The main characteristics of each study are shown in Table 2. All studies were retrospective analyses and provided specific inclusion and/or exclusion criteria. The number of studies using DSA to demonstrate the BSO sign (3) was approximately equal to that of CTA (4).

**Table 2:** Study characteristics.

**References**	**Year**	**Type**	**Location**	**Radiography**	**BSO (*n*)**	**TP (*n*)**	**FP (*n*)**	**FN (*n*)**	**TN (*n*)**	**Sensitivity (95% CI)**	**Specificity (95% CI)**
Baek et al. (9)	2016	Retro	ICA	DSA	227	201	26	15	17	0.93	0.40
			MCA							(0.89–0.96)	(0.25–0.56)
			BA								
Pikija et al. (14)	2017	Retro	ICA	CTA	18	14	4	7	2	0.67	0.33
										(0.43–0.85)	(0.04–0.78)
Lee et al. (8)	2019	Retro	ICA	CTA	162	137	25	14	5	0.91	0.17
			MCA							(0.85–0.95)	(0.06–0.35)
			BA								
Ota et al. (17)	2019	Retro	ICA	DSA	57	53	4	13	3	0.80	0.43
										(0.69–0.89)	(0.10–0.82)
Lee et al. (5)	2020	Retro	MCA	CTA	252	200	52	14	24	0.93	0.32
												(0.89–0.96)	(0.21–0.43)
Chuming et al. (13)	2020	Retro	ICA	DSA	69	66	3	7	39	0.90	0.93
			MCA							(0.81–0.96)	(0.81–0.99)
			BA								
Lee et al. (18)	2020	Retro	BA	CTA	32	29	3	10	23	0.74	0.88
												(0.58–0.87)	(0.70–0.98)
Chen et al. (3)	2020	Retro	MCA	DSA	51	51	0	9	20	0.85	1.00
											(0.73–0.93)	(0.83–1.00)

Retro, retrospective analysis; ICA, internal carotid artery; MCA, middle cerebral artery; BA, basilar artery; DSA, digital subtraction angiography; CTA, computed tomography angiography; BSO, branching site occlusion; TP, true positive; FP, false positive; TN, true negative; FN, false negative; 95% CI, 95% confidence interval.

### 3.3. Quality assessment

**Figure 2:**
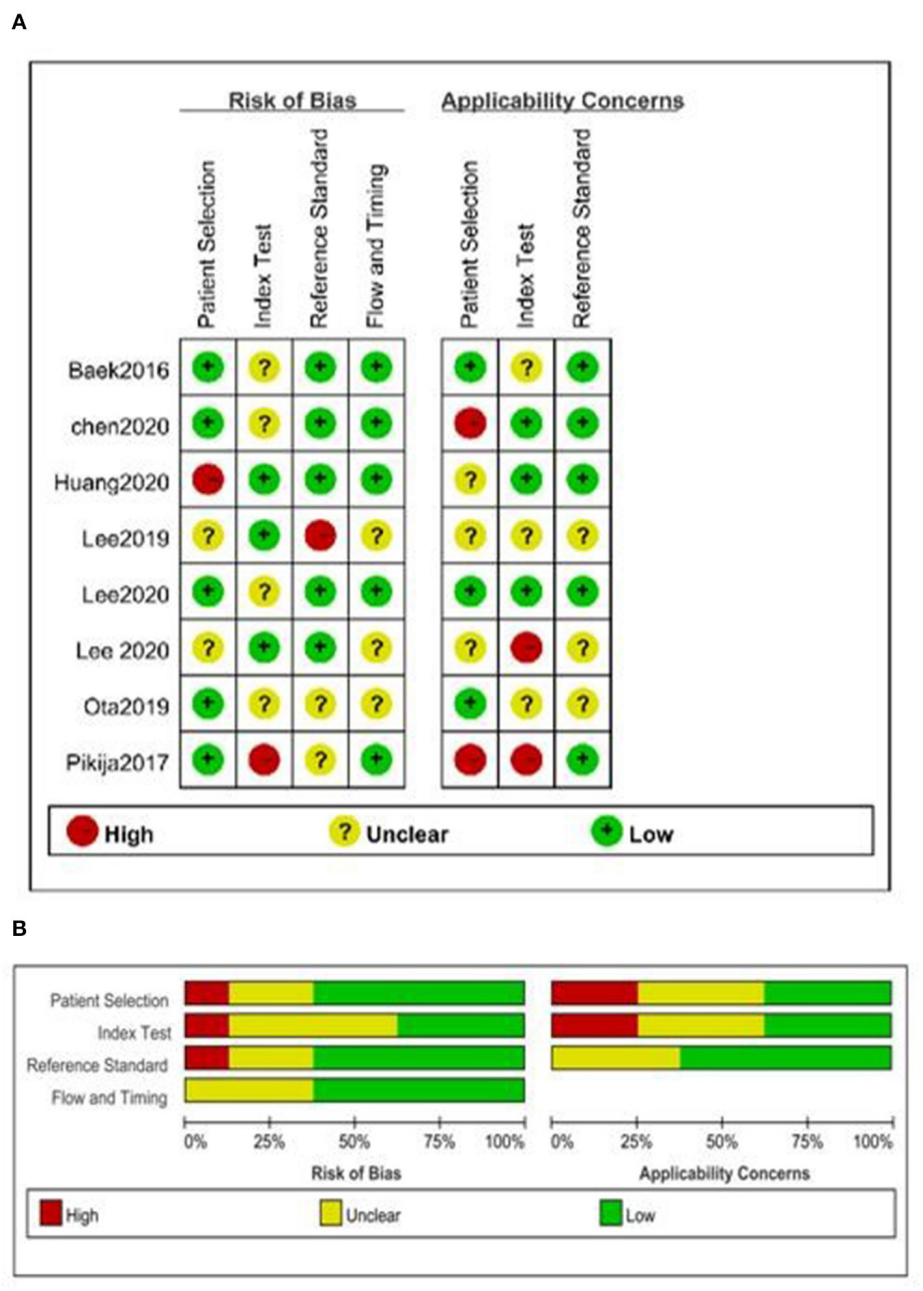
Quality assessment of included studies. **(A)** Summary of risk of bias and applicability concerns and **(B)** risk of bias and applicability concerns graph.

The overall quality was moderate (Figure 2). None of the included studies fulfilled all the items of the QUADAS-2 checklist. Some studies did not report whether a consecutive or random sample of patients was enrolled (8, 13, 18); two studies did not mention whether the BSO sign was interpreted without knowledge of the results of the reference standard, and it was unclear if reviewers of the reference standard were blinded to the results of TTO sign (Figure 2) (8, 14).

### 3.4. Meta-analysis

#### 3.4.1. The predictive value of the BSO sign for predicting ILVO-AIS etiology

**Figure 3:**
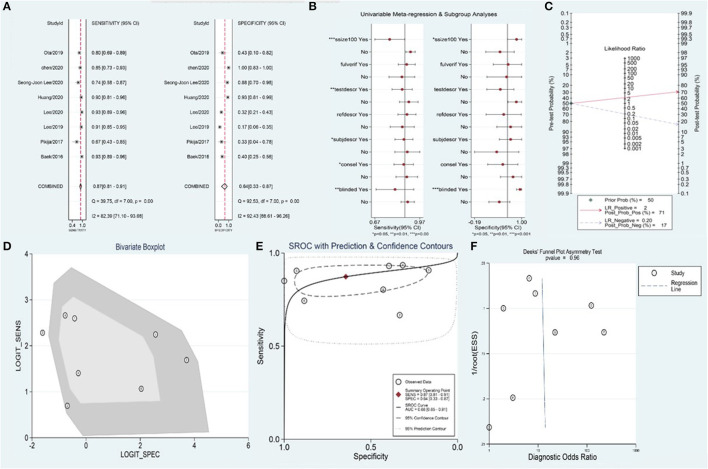
Meta-analysis of BSO for diagnosis of embolism-AIS. **(A)** Sensitivity and specificity of BSO for the diagnosis of embolism-AIS, **(B)** meta-regression of the BSO for diagnosis of embolism-AIS, **(C)** Fagan nomogram of the BSO for diagnosis of embolism-AIS, **(D)** bivariate boxplot of the BSO for diagnosis of embolism-AIS, **(E)** summary receiver operating characteristic (ROC) curve of BSO for diagnosis of embolism-AIS, and **(F)** funnel plot of publication bias about BSO diagnosis of embolism-AIS.

A total of 1,090 patients were included in the meta-analysis for predicting the value of the BSO sign for embolism-AIS. Of these, 868 patients had a BSO sign evident by DSA or CTA, 627 patients of whom were finally diagnosed with embolism-AIS; the remaining cases were diagnosed as ICAS-AIS. Summary estimates of sensitivity and specificity by random-effects were 0.87 (95% CI 0.81–0.91) and 0.64 (95% CI 0.33–0.87), respectively (Figure 3A). The area under the curve was 0.88 (95% CI 0.85–0.91) (Figure 3E). The positive likelihood ratio was 2.5 (95% CI 1.1–5.7) (a person diagnosed with embolism-AIS by BSO sign is 2.5 times more likely than non-embolism), the negative likelihood ratio was 0.20 (95% CI 0.12–0.33), and the diagnostic odds ratio was 12 (95% CI 3–44). In patients with suspected AIS, in the case of 50% pre-test probability, the post-test probability of a positive test result was 71% (Figure 3C).

The overall *I*^2^ for the bivariate model was 97% (95% CI 0.95–0.99%), suggesting there was substantial heterogeneity between studies (Figure 3D). Meta-regression indicated that the heterogeneity of sensitivity mainly came from the sample size, blinding, and the consistency of index test standard and reference standard of included studies. Additionally, the heterogeneity of specificity mainly came from the sample size and blinding of included studies (Figure 3B). No significant publication bias was observed in the included studies (*p* = 0.96) (Figure 3F).

#### 3.4.2. Therapeutic outcomes

A total of 517 patients were included in the meta-analysis evaluating the predictive value of the BSO sign for RS after SR without RT. RS after SR without RT was achieved in 495 patients, and the BSO sign was found in 295 of these cases.

**Figure 4:**
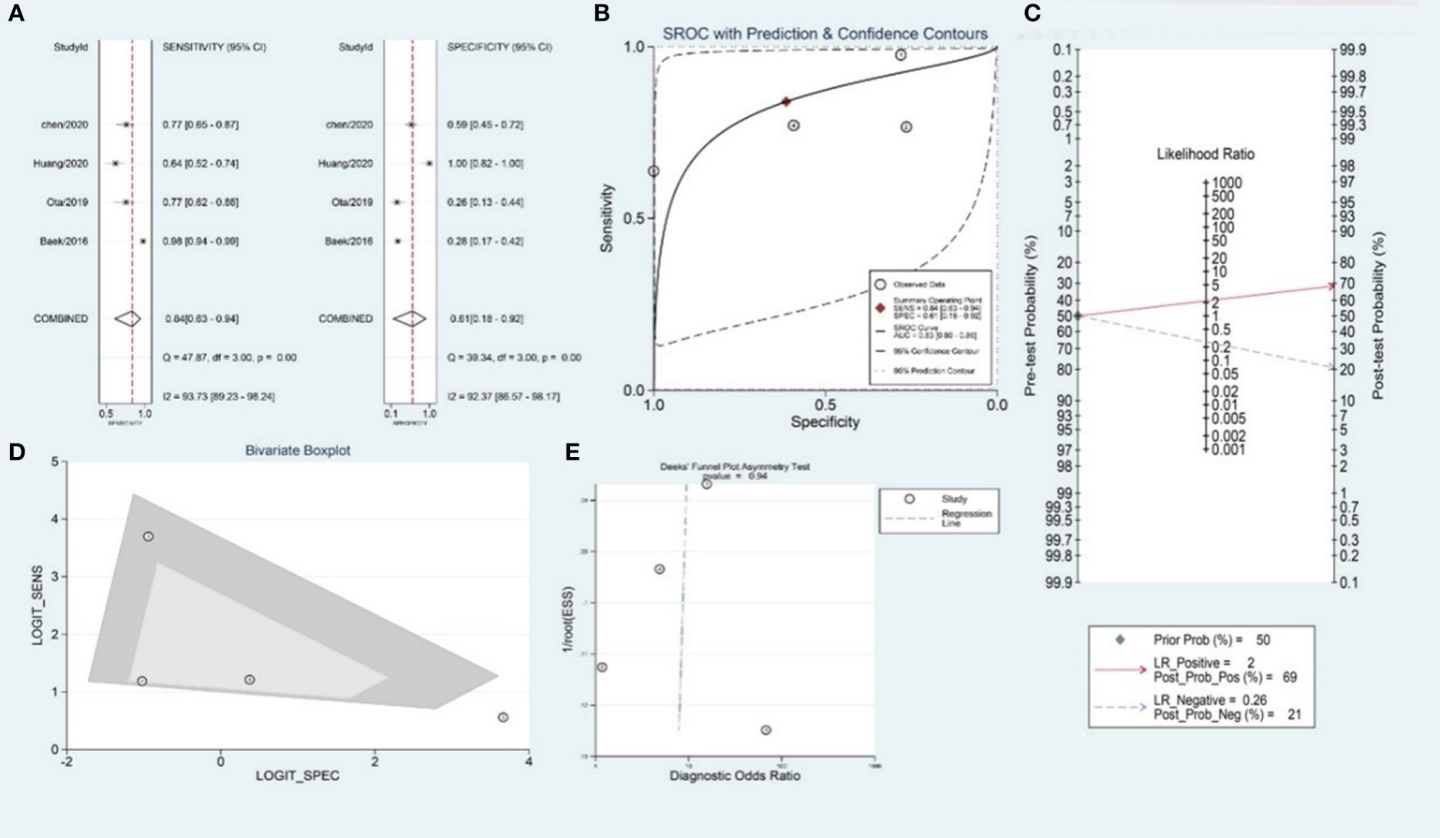
Meta-analysis of BSO for the prediction of RS after SR without rescue therapy. **(A)** Sensitivity and specificity of BSO for the prediction of RS after SR without RT, **(B)** summary ROC curve of BSO for the prediction of RS after SR without RT, **(C)** Fagan nomogram of the BSO for the prediction of RS after SR without RT, **(D)** bivariate boxplot of BSO for the prediction of RS after SR without RT, and **(E)** funnel plot of publication bias about BSO for the prediction of RS after SR without RT.

The pooled sensitivity and specificity were 0.84 (95% CI 0.63–0.94) and 0.61 (95% CI 0.18–0.92), respectively (Figure 4A). The area under the curve was 0.83 (95% CI 0.80–0.86) (Figure 4B). The positive likelihood ratio was 2.2 (95% CI 0.7–6.6), the negative likelihood ratio was 0.26 (95% CI 0.12–0.58), and the diagnostic odds ratio was 8 (95% CI 2.0–4.0). In patients with suspected RF after primary thrombectomy, with a pre-test probability of 50%, the post-test probability of positive test results was 69% (Figure 4C).

The overall *I*^2^ for the bivariate model was 97% (95% CI 95–99), suggesting there was substantial heterogeneity between studies (Figure 4D). To further determine the source of heterogeneity, we planned to perform a meta-regression analysis, but it could not be achieved due to the small number of included studies. No significant publication bias was observed in the included studies (*p* = 0.94) (Figure 4E).

## 4. Discussion

Stentriever thrombectomy is the main EVT technique used for ILVO-AIS (19). However, it is ineffective in ~20% of patients (20, 21). It is thought that the success of SR is associated with the pathogenesis of ILVO-AIS (10). Non-embolic ILVO-AIS resulting from intracranial atherosclerotic stenosis tends to get less benefit from SR. Therefore, the pre-procedural identification of ILVO-AIS etiology may predict the effect of SR and help operators choose the optimal EVT strategy to increase RS rates and shorten puncture-to-recanalization times (9).

Several methods, including the hyperdense artery sign, susceptibility vessel sign, fixed focal stenosis on follow-up imaging, histological examination of extracted clots, comprehensive cardiological evaluation after the lapse of the hyperacute stage, and intra-procedural angiographic signs (cut-off, tapered, and meniscus) could predict the pathogenesis of ILVO-AIS and response to first-line endovascular strategies (7). However, several studies have reported the relationship between the BSO sign and the etiology of ILVO-AIS (9, 14). It was thought that the BSO sign could be an independent predictor of the underlying stroke mechanism and SR response. In actuality, the concept of the BSO sign is related to the generation of embolic-ILVO-AIS. An embolus or floating clot has a greater tendency to obstruct an arterial branching site, while on the contrary, ICAS seems to predominantly involve arterial trunks before bifurcation (22). In this study, we conducted a meta-analysis to evaluate the sensitivity and specificity of the BSO sign as an imaging marker of embolic ILVO-AIS. We found that the BSO sign could be a valid and precise marker to predict embolic ILVO-AIS, with a sensitivity of 87% and a specificity of 64%.

Patients with ILVO-AIS from different causes responded differently to SR treatment. In cases resulting from ICAS, very high re-occlusion rates were present after initial recanalization by RS. This resulted in prolonged puncture-to-recanalization time and a worse prognosis. The use of an SR might cause atheromatous surface injury, thus increasing platelet activation and leading to re-occlusion and even dissection. On the contrary, AIS caused by embolism was highly responsive to SR-based EVT and was associated with a very low re-occlusion rate. Previous studies have shown that patients with embolic occlusion treated with SR presented with both higher recanalization rates and better prognoses (23–25). Embolism-AIS is commonly caused by atrial fibrillation, which usually consists of red thrombi that are rich in erythrocytes (26). The characteristics of the red thrombi were higher clot burden and higher density than white thrombi (6, 27). These physiological characteristics may be associated with the successful SR (6, 9). However, on the contrary, previous studies showed that thrombi from a large atherosclerotic artery had the highest volume fraction of RBCs, while cardioembolic thrombi had the lowest (28). Further studies are needed for revealing the association between the etiology and the pathogenesis of cerebral thrombi to improve the efficacy of choosing first-line endovascular strategies and clinical outcomes (29).

In this context, the identification of ILVO-AIS etiology is critical for EVT strategy choice, which might influence the recanalization success rate and patient prognosis. Our results showed that patients with embolic-AIS treated by SR had favorable clinical outcomes. Additionally, the BSO sign could be a valid and precise imaging marker to predict RS by SR without RT with a sensitivity of 84% and a specificity of 61%.

### 4.1. Conclusion

Our meta-analysis found that the BSO sign could be a valid and precise imaging marker to predict embolic ILVO-AIS and RS by SR without RT. This may provide potential evidence for choosing a first-line EVT strategy to increase the RS rate and shorten puncture-to-recanalization time.

## Data availability statement

The original contributions presented in the study are included in the article/supplementary material, further inquiries can be directed to the corresponding authors.

## Author contributions

XJ drafting and revision of the manuscript for content, including medical writing for content, study concept or design, and analysis or interpretation of data. ZS drafting and revision of the manuscript for content, including medical writing for content, major role in the acquisition of data, and analysis or interpretation of data. PW major role in the acquisition of data. XW and FL drafting and revision of the manuscript for content, including medical writing for content, major role in the acquisition of data, study concept or design, and analysis or interpretation of data. All authors contributed to the article and approved the submitted version.
